# Single Neuronal Dynamical System in Self-Feedbacked Hopfield Networks and Its Application in Image Encryption

**DOI:** 10.3390/e23040456

**Published:** 2021-04-13

**Authors:** Xitong Xu, Shengbo Chen

**Affiliations:** College of Geo-Exploration Science and Technology, Jilin University, Changchun 130026, China; xitong19@mails.jlu.edu.cn

**Keywords:** single neuronal dynamical system, self-feedbacked, hopfield network, chaos, image encryption

## Abstract

Image encryption is a confidential strategy to keep the information in digital images from being leaked. Due to excellent chaotic dynamic behavior, self-feedbacked Hopfield networks have been used to design image ciphers. However, Self-feedbacked Hopfield networks have complex structures, large computational amount and fixed parameters; these properties limit the application of them. In this paper, a single neuronal dynamical system in self-feedbacked Hopfield network is unveiled. The discrete form of single neuronal dynamical system is derived from a self-feedbacked Hopfield network. Chaotic performance evaluation indicates that the system has good complexity, high sensitivity, and a large chaotic parameter range. The system is also incorporated into a framework to improve its chaotic performance. The result shows the system is well adapted to this type of framework, which means that there is a lot of room for improvement in the system. To investigate its applications in image encryption, an image encryption scheme is then designed. Simulation results and security analysis indicate that the proposed scheme is highly resistant to various attacks and competitive with some exiting schemes.

## 1. Introduction

Neural networks and neuro-dynamics expand to different application areas including signal processing, information security, encryption and associative memory [1,2,3,4,5,6]. The Hopfield network is a typical dynamic neural network with abundant dynamic characteristics. Since Hopfield proposed the model, it has been applied to solving multifarious optimization problems [7,8,9]. However, the conventional Hopfield network often obtained a solution which was far from the optimal solution [10].

Since the obstacle was reported, multitudinous improved methods have been applied to the Hopfield network [11,12,13,14,15]. Among these modifications of the Hopfield network, a self-feedbacked Hopfield network has similar properties with the conventional Hopfield network, but have higher convergence speed [15]. It was also proved to have good chaotic dynamic behavior [16]. Therefore, the self-feedbacked Hopfield network has been widely used in optimization problems and image encryption [17,18,19,20,21,22,23,24]. However, the self-feedbacked Hopfield network still has some interesting properties to be discovered. We found that the single neuron of the self-feedbacked Hopfield network also showed complex dynamic behavior. Self-feedbacked Hopfield networks that were used to generate chaos phenomena have complex structures, a large computational amount and fixed parameters [16,18,19,20,21,22]. Due to these properties, self-feedbacked Hopfield networks need to be combined with other chaotic maps [18,19,20,21], which have consequently limited the application. On the contrary, the structure and calculation of single neuron are simplified, and the single neuron can present chaos phenomenon as its parameters vary in continuous range. Therefore, the single neuron has a broad application scope.

In recent years, chaotic systems have been widely applied in cryptography and pseudo-random number [25,26,27,28,29]. The orbits of high-dimensional chaotic systems are difficult to be predicted, but the systems need complex performance analysis and high implementation costs [30,31]. Many simple chaotic systems (e.g., logistic map, sine map, and chebyshev map) have been used to achieve high efficiency due to fewer parameters and a simple structure [32,33,34,35,36,37,38,39]. However, the simple chaotic systems have drawbacks in the application. Due to the small number of parameters, the key space is limited, and the initial states can be estimated through certain methods [40,41,42]. This makes the applications of simple chaotic systems not secure enough [41,43,44]. Also, sensitivity to the effects of computer precision may degenerate the systems into being non-chaotic immediately [45]. The single neuronal dynamical system in a self-feedbacked Hopfield network has sufficient parameters and excellent chaotic properties.

In addition, various frameworks that can improve the properties of simple chaotic systems have been proposed, including a combination of multiple maps [46,47,48,49], modifying the chaotic sequences generated by chaotic maps [50,51,52], and modifying the existing maps [53,54,55]. Most of the frameworks set the existing maps as a whole and incorporate them into the fixed format [54,56,57,58], thus generating new maps with better performance automatically. The single neuronal dynamical system in self-feedbacked Hopfield network is also applicable to existing frameworks, and it can achieve a positive effect.

In this paper, the discrete form of single neuronal dynamical system (SNDS) is derived from the self-feedbacked Hopfield network. Moreover, SNDS is incorporated into a framework and enhanced single neuronal dynamical system (ESNDS) is produced. At last, an image encryption scheme based on the ESNDS is designed.

The paper is organized as follows. Section 2 presents the derivation process of the SNDS discrete form. Section 3 demonstrates the chaotic dynamic behavior of SNDS from two perspectives. The structure and performance of ESNDS is shown in Section 4, where the sequences generated from ESNDS are also test. Section 5 shows an encryption scheme and the results of simulation and analysis. Section 6 reveals our conclusion.

## 2. Mathematical Preliminaries

### 2.1. The Hopfield Networks

**Figure 1:**
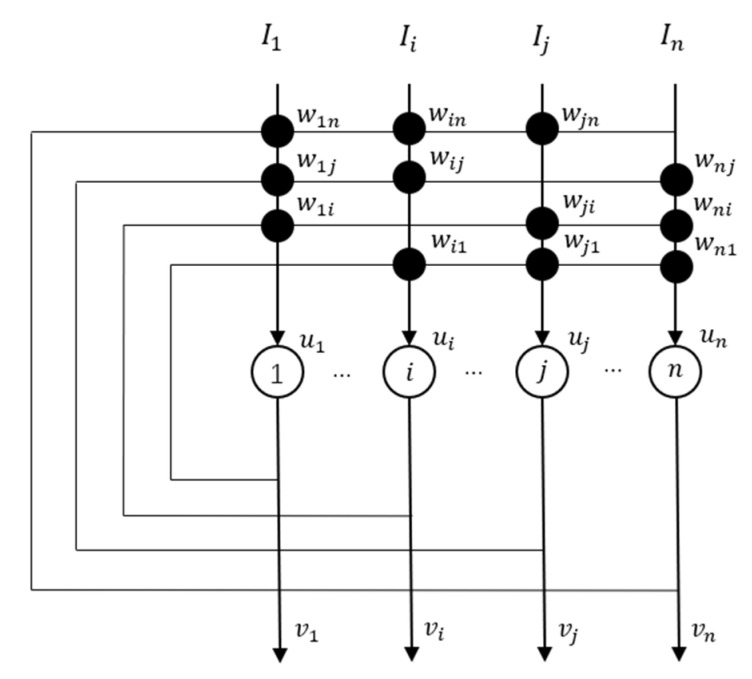
The structure of conventional Hopfield network [8].

The Hopfield network [8] is defined as Equations (1) and (2):

(1) $$v_{i}\left(t\right)=f\left[u_{i}\left(t\right)\right],$$

(2) $$C_{i}\frac{du_{i}\left(t\right)}{dt}=-\frac{u_{i}\left(t\right)}{R_{i}}+\sum_{j=1}^{n}T_{ij}v_{j}\left(t\right)+I_{i},$$

where, $C_{i}$ represents the capacitance of the i neuron; $u_{i}\left(t\right)$ represents the input of neuron i at time instance, t; $v_{j}\left(t\right)$ represents the output of neuron j at time instance, t; $R_{i}$ is the resistance of neuron i; $T_{ij}^{-1}$ is the finite impedance between the output $v_{j}$ and the neuron i; $I_{i}$ is any other fixed input current to neuron i; f() is the activation function of neurons. The structure of conventional Hopfield network is shown in Figure 1.

We assume $\varepsilon_{i}=R_{i}C_{i}$, $w_{ij}=R_{i}T_{i}$, $b_{i}=R_{i}I_{i}$. Also, assuming that $u\left(t\right)={\left[u_{1}\left(t\right),u_{2}\left(t\right),\ldots ,\;u_{n}\left(t\right)\;\right]}^{T}$, $v\left(t\right)={\left[v_{1}\left(t\right),v_{2}\left(t\right),\ldots ,v_{n}\left(t\right)\right]}^{T}$, $\varepsilon =Diag\left(\varepsilon_{1},\varepsilon_{2},\ldots ,\varepsilon_{n}\right)$. According to Figure 1, there is no self-feedbacks in conventional Hopfield Neural network, we can denote $w=[w_{ij}]$ in the condition of $w_{ij}=w_{ji}$ and $w_{ii}=0$, then Equations (1) and (2) are transformed to Equations (3) and (4):

(3) v(t)=f[u(t)],

(4) $$\varepsilon \frac{du\left(t\right)}{dt}=-u\left(t\right)+Wv\left(t\right)+b.$$

### 2.2. Single Neuronal Dynamical System in Self-Feedbacked Hopfield Networks

**Figure 2:**
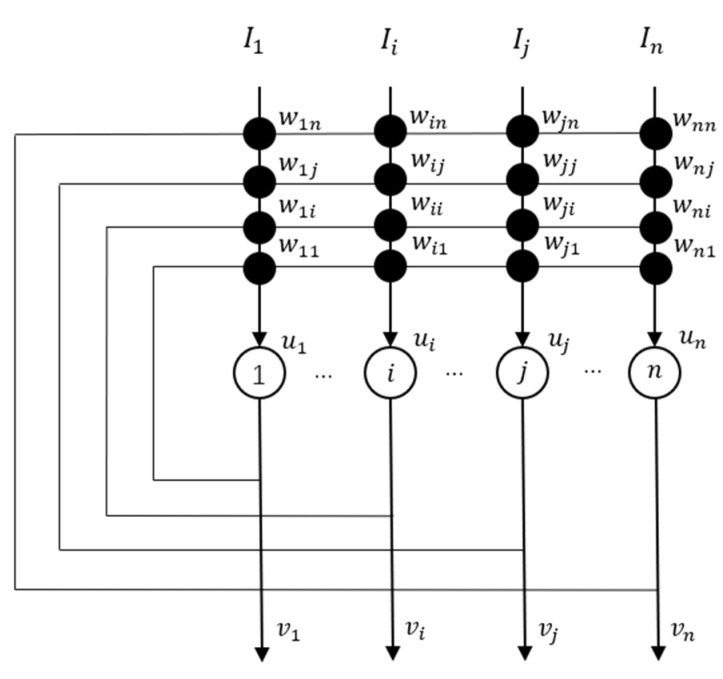
The structure of positively self-feedbacked Hopfield network [15].

The structure of self-feedbacked Hopfield network is shown in Figure 2.

According to Figure 2, we have $w_{ii}\neq 0$. For a single neuron, we don’t add the output of other neurons, so set $w_{ij}=0$, j∈[1,2,…,n] (j≠i). The Equations (3) and (4) can be converted into single neuron format, as Equations (5) and (6):

(5) $$v_{i}\left(t\right)=f\left(u_{i}\left(t\right)\right),$$

(6) $$\frac{u_{i}\left(t+\Delta t\right)-u_{i}\left(t\right)}{\Delta t}=-\frac{u_{i}\left(t\right)}{\varepsilon_{i}}+\frac{1}{\varepsilon_{i}}w_{ii}v_{i}\left(t\right)+\frac{b_{i}}{\varepsilon_{i}},$$

where, Δt is unit interval, set Δt=1. The Equation (7) can then be obtained:

(7) $$u_{i}\left(t+1\right)=\left(1-\frac{1}{\varepsilon_{i}}\right)u_{i}\left(t\right)+\frac{1}{\varepsilon_{i}}w_{ii}v_{i}\left(t\right)+\frac{1}{\varepsilon_{i}}b_{i}.$$

We assume $k=1-\frac{1}{\varepsilon_{i}}$, $z=\frac{1}{\varepsilon_{i}}w_{ii}$, $h=\frac{1}{\varepsilon_{i}}b_{i}$, then Equation (7) is transformed to Equation (8):

(8) $$u_{i}\left(t+1\right)=ku_{i}\left(t\right)+zv_{i}\left(t\right)+h.$$

For conventional Hopfield network, the activation function is sigmoid. Therefore, this study uses sigmoid function as activation function. The Equation (5) is transformed to Equation (9):

(9) $$v_{i}\left(t\right)=\frac{1}{1+exp\left(-\gamma u_{i}\left(t\right)\right)}.$$

Thus, the single neuronal dynamical system in self-feedbacked Hopfield networks is obtained.

## 3. Analysis of Single Neuronal Dynamical System

### 3.1. Dynamical Behavior in Single Neuronal Dynamical System

**Figure 3:**
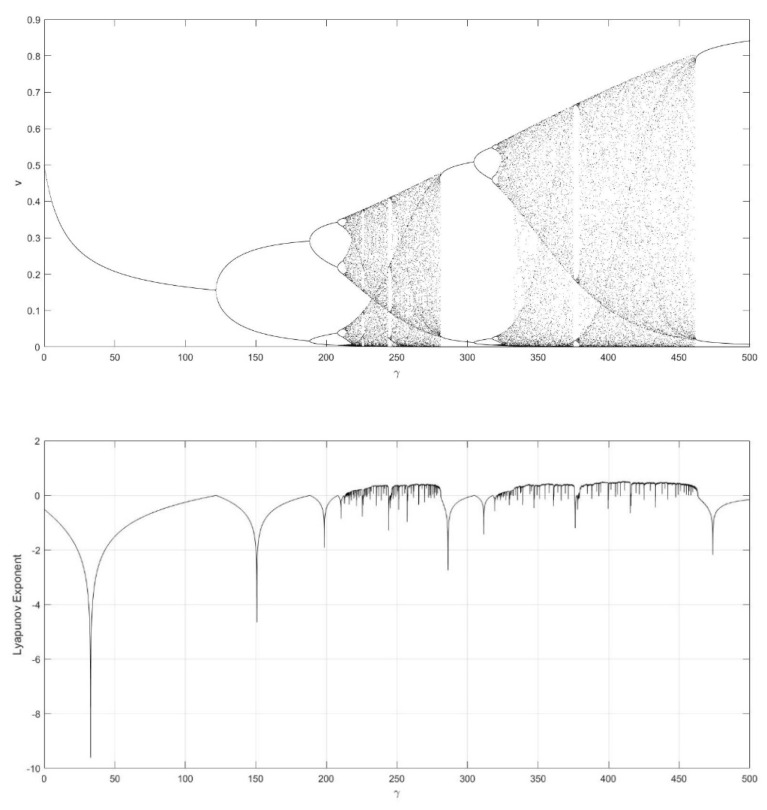
Single-parameter bifurcation diagram of v versus parameter γ and corresponding Lyapunov exponent diagram for k=0.6, z=−0.1, and h=0.01.

**Figure 4:**
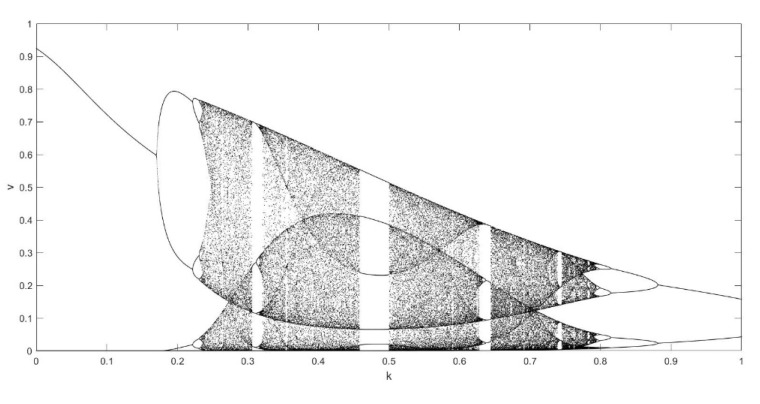

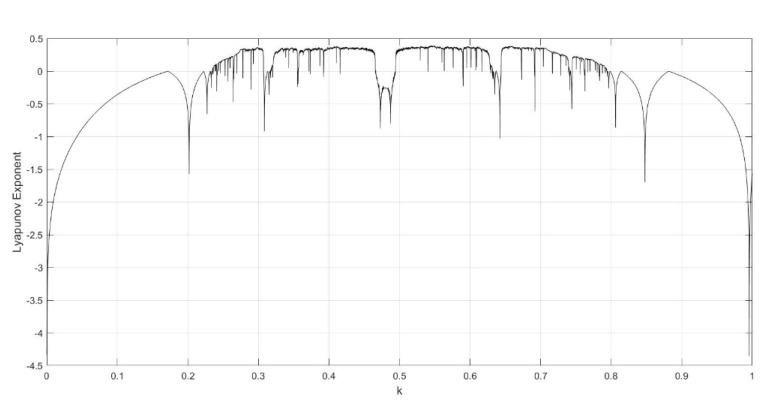
Single-parameter bifurcation diagram of v versus parameter k and corresponding Lyapunov exponent diagram for γ=250, z=−0.1, and h=0.01.

**Figure 5:**
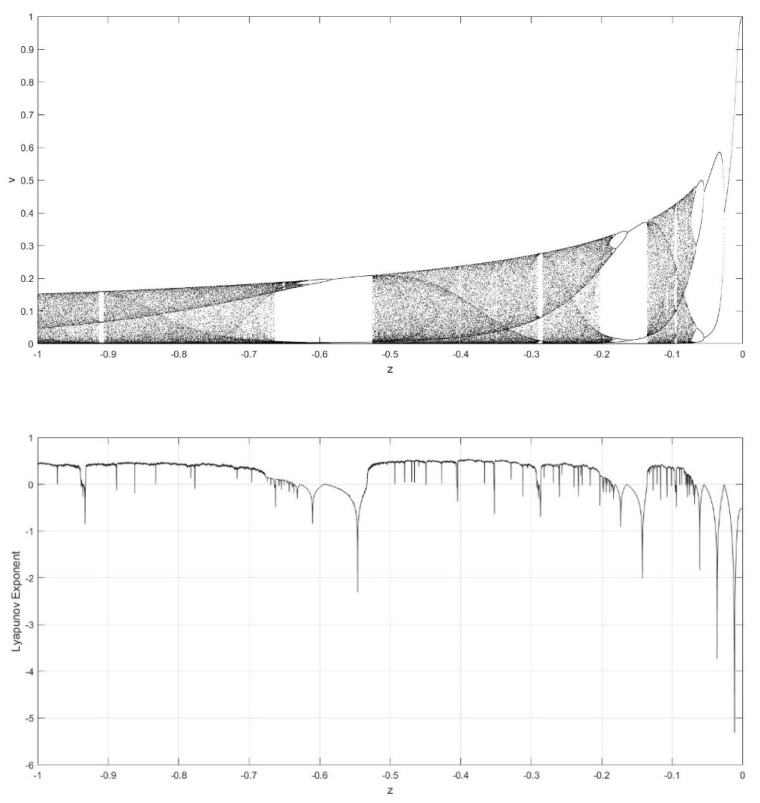
Single-parameter bifurcation diagram of v versus parameter z and corresponding Lyapunov exponent diagram for γ=250, k=0.6, and h=0.01.

**Figure 6:**
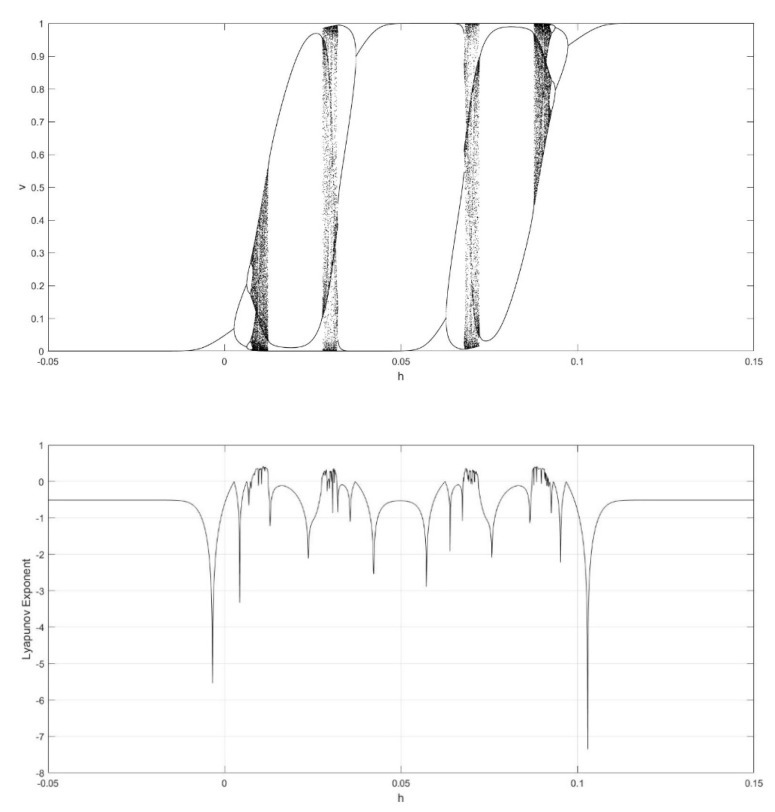
Single-parameter bifurcation diagram of v versus parameter h and corresponding Lyapunov exponent diagram for γ=250, k=0.6, and z=−0.1.

On the basis of Equations (8) and (9), it should be noted that the single neuronal dynamical system (SNDS) has four parameters. We can vary them to show complex dynamic behaviors. When the parameters hold specific value, a sequence of bifurcation leading to chaos can be observed by changing one parameter. To unmask the dynamical behavior of the SNDS, the single-parameter bifurcation diagrams and the corresponding evolution diagrams of the Lyapunov exponent are drawn, as shown in Figure 3, Figure 4, Figure 5 and Figure 6. In the figures, there is distinct correspondence between bifurcation diagrams and evolution diagrams of the Lyapunov exponent. For parameter γ, Figure 3 shows multiple instances of entering and exiting chaos, which are associated with multiple bifurcations phenomenon. The instances that exit chaos are sudden, and it corresponds to the sudden decrease of Lyapunov exponent in the evolution diagram. For parameter k, as shown in Figure 4, it first gradually enters chaos, and then gradually exits after a period of evolution. In the evolution, chaos is not continuous. Furthermore, the Lyapunov exponent diagram of k has symmetry in the domain of definition. Parameter z also appears discontinuous chaos phenomenon in a large range, and parameter h appears chaos phenomenon only within a very small range.

**Figure 7:**
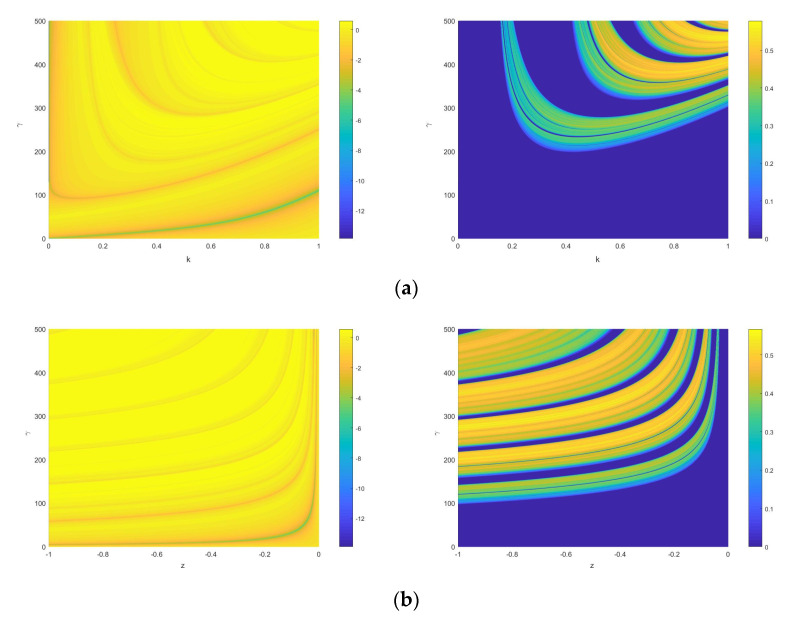

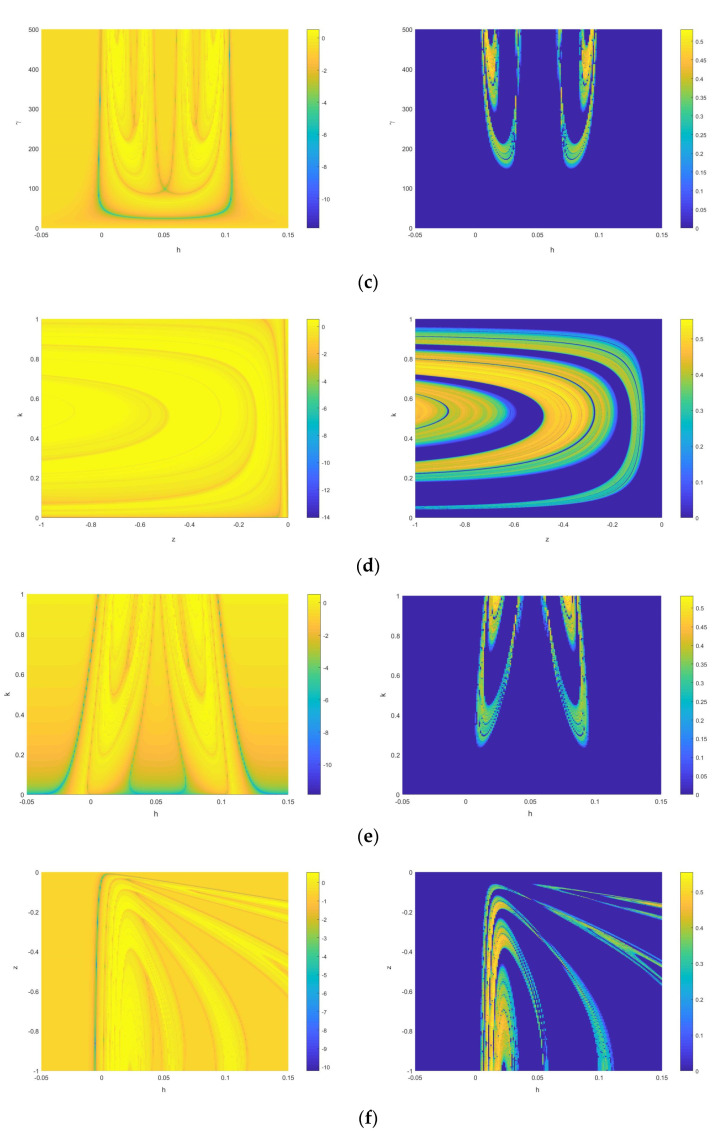
Two-dimensional evolution diagram of Lyapunov exponent (the original), and two-dimensional evolution diagram of Lyapunov exponent (set Lyapunov exponent<0 to 0 ) of (**a**) k -γ for z=−0.1 and h=0.01; (**b**) z -γ for k=0.6 and h=0.01; (**c**) h -γ for k=0.6 and z=−0.1; (**d**) z -k for γ=250 and h=0.01; (**e**) k -h for γ=250 and z=−0.1; (**f**) z -h for k=0.6 and γ=250.

In addition, the double-parameter evolution diagrams of Lyapunov exponent are used for a clearer understanding of the dynamical behavior of SNDS, as shown in Figure 7. The Figure includes six parameter combinations. Each combination is presented by two two-dimensional evolution diagrams of Lyapunov exponent. The latter two-dimensional evolution diagram is formed on the basis of setting Lyapunov exponent which is less than zero to be zero. In Figure 7, some interesting phenomenon can be observed. The combinations of γ−z, γ−k, and k−z appear wide area of chaos, and the area is banded in the diagram. This corresponds to the single-parameter evolution diagram of the Lyapunov exponent. On the contrary, the chaos range of the combination with parameter h is narrow.

### 3.2. Efficiency Analysis

High efficiency of the chaotic map is necessary as practical applications always involve the generation of a large number of pseudorandom sequences. Compared with self-feedbacked Hopfield networks, SNDS has low implementation cost. Table 1 shows the time elapsed by SNDS and self-feedbacked Hopfield networks when generating pseudorandom sequences. The experimental environments are as follows: Matlab R2017a, Intel (R) Core (TM) i5-9400F CPU @ 2.90 GHz with 24 GB memory, Windows 10 Operation System. In the experiment, each sequence is generated 100 times, and the average running time is taken as the result. This indicates that SNDS has the higher efficiency than self-feedbacked Hopfield networks.

## 4. Enhanced Single Neuronal Dynamic System and Random Bit Generation

### 4.1. Enhanced Single Neuronal Dynamic System

**Figure 8:**
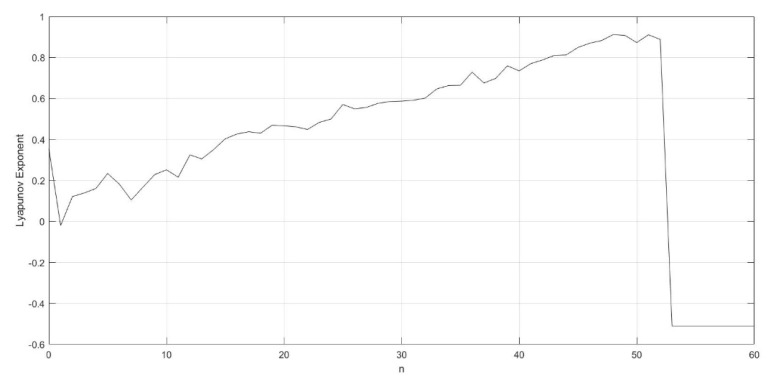
The evolution diagram of Lyapunov exponent versus parameter n for γ=250, k=0.6, z=−0.1, and h=0.01.

By incorporating SNDS into the framework proposed in [54], The enhanced single neuronal dynamic system (ESNDS) is obtained. It is described by Equation (10):

(10) $$\{\begin{array}{c} v_{i}\left(t\right)=\frac{1}{1+exp\left(-\gamma u_{i}\left(t\right)\right)}\\ v_{i}^{\prime }\left(t\right)=v_{i}\left(t\right)\times 2^{n}-floor\left(v_{i}\left(t\right)\times 2^{n}\right)\\ u_{i}\left(t+1\right)=ku_{i}\left(t\right)+zv_{i}^{\prime }\left(t\right)+h \end{array},$$

where the parameter $v_{i}^{\prime }\left(t\right)$ is the value of $v_{i}\left(t\right)$ after an intermediate calculation. The Lyapunov exponent evolution diagram of n is shown as Figure 8. In this paper, n is set to a fixed value of 14.

**Figure 9:**
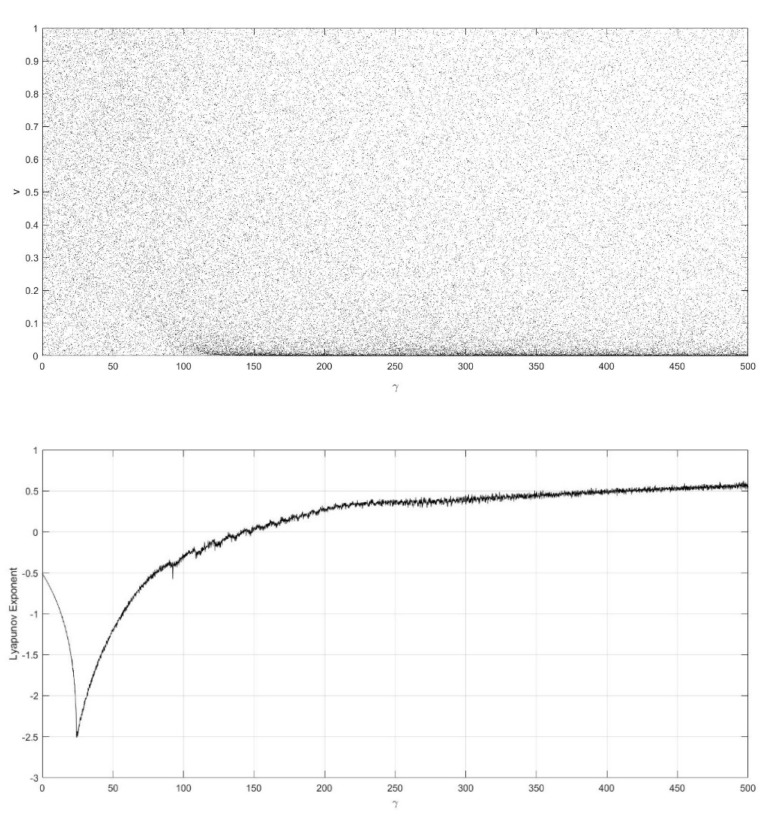
Single-parameter bifurcation diagram of v versus parameter γ and corresponding Lyapunov exponent diagram for k=0.6, z=−0.1, and h=0.01.

**Figure 10:**
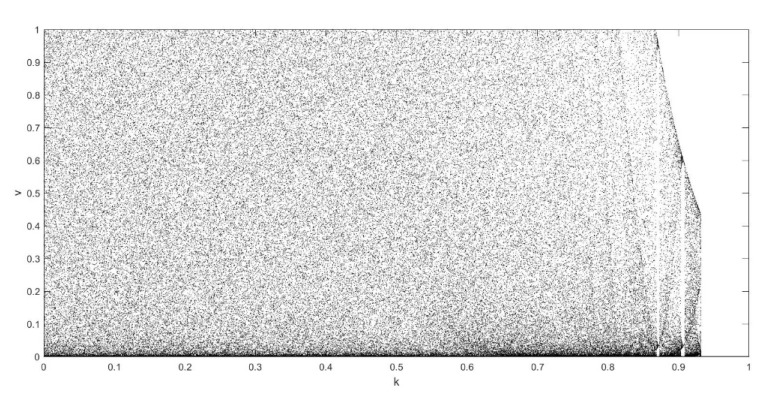

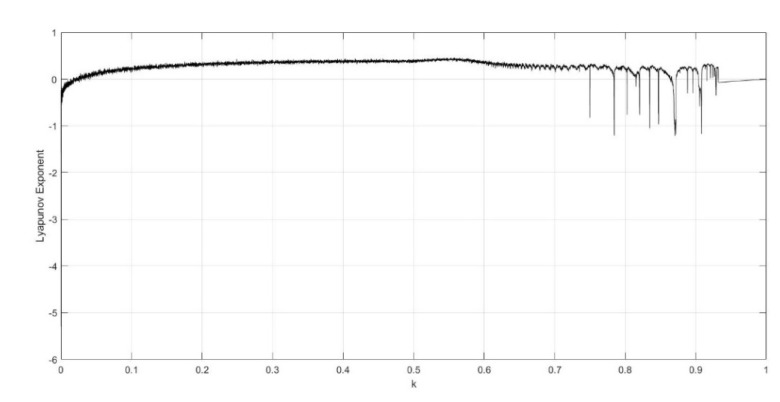
Single-parameter bifurcation diagram of v versus parameter k and corresponding Lyapunov exponent diagram for γ=250, z=−0.1, and h=0.01.

**Figure 11:**
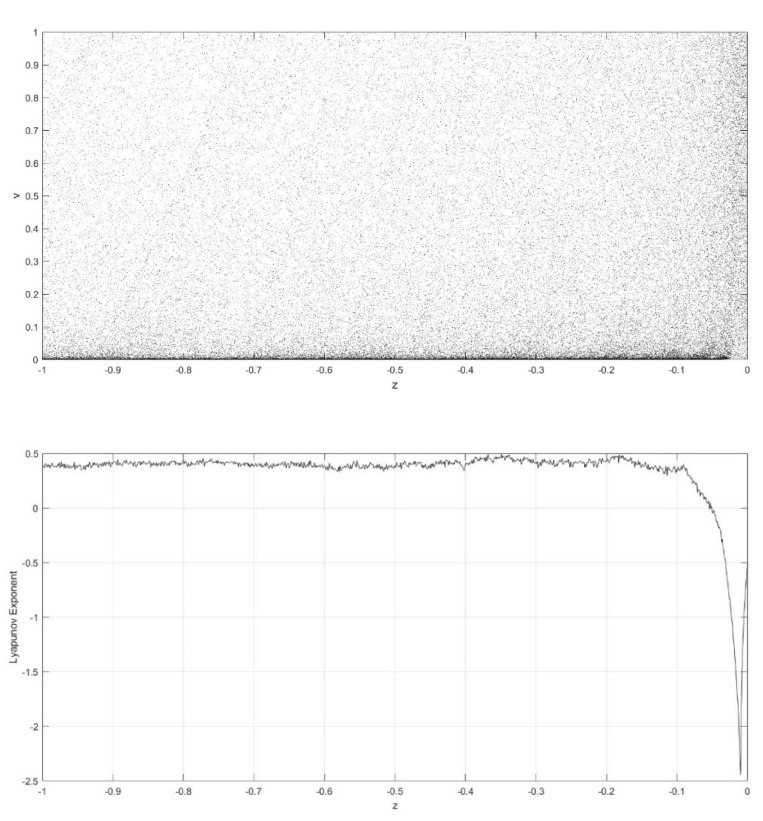
Single-parameter bifurcation diagram of v versus parameter z and corresponding Lyapunov exponent diagram for γ=250, k=0.6, and h=0.01.

**Figure 12:**
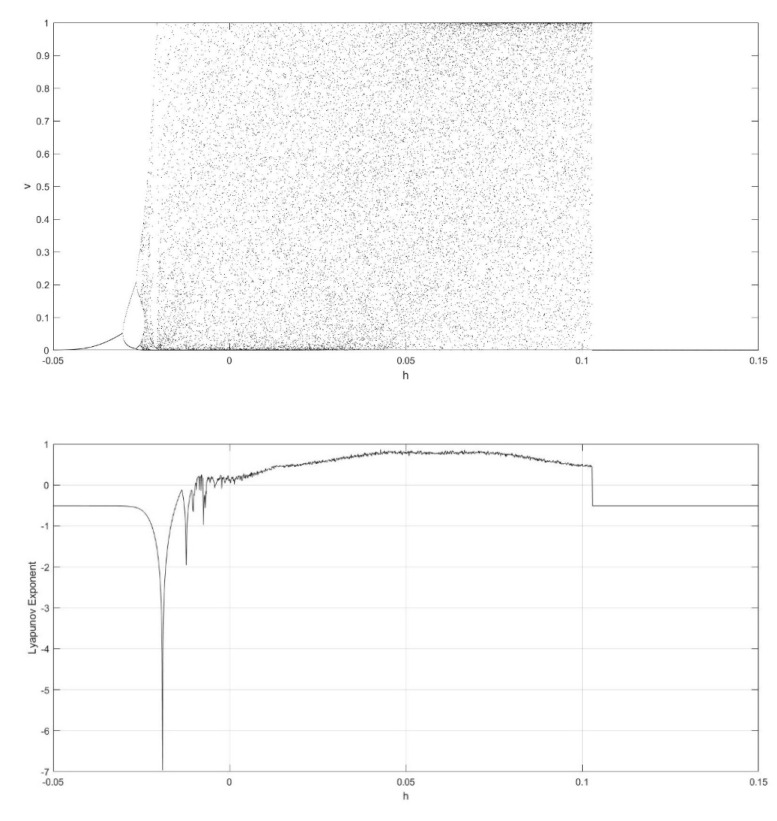
Single-parameter bifurcation diagram of v versus parameter h and corresponding Lyapunov exponent diagram for γ=250, k=0.6, and z=−0.1.

For ESNDS, the bifurcation diagrams and Lyapunov exponent evolution diagrams of single-parameter are shown in Figure 9, Figure 10, Figure 11 and Figure 12. It can be seen that the chaotic range of all parameters tends to be continuous. The Lyapunov exponent of Parameter γ falls first and rises later, and Lyapunov exponent>0 occurs around γ=150. The other three parameters are also in chaos over a wide range. Note that for h, the chaotic property of this parameter has been greatly improved. It means that SNDS can achieve better performance by using the frameworks suitable for a simple chaotic system. This greatly increases the application potential of SNDS.

### 4.2. Random Bit Generation

#### 4.2.1. NIST SP800-22 Test

To demonstrate the robustness of ESNDS and the potential of its application in image encryption, the NIST SP800-22 test standard is used for ESNDS. It is designed by National Institute of Standards and Technology (NIST) to validate the randomness of binary sequences [59]. NIST SP800-22 is the most complete statistical test suite for randomness test of binary sequences [60]. The binary numbers are generated by the value of $v_{i}^{\prime }$ in the iterative process of ESNDS. For each value of $v_{i}^{\prime }$, we discard the former 10 decimal digits and compare the result with 0.5, the process is shown as Equations (11) and (12):

(11) $$s_{i}=(10^{10}\times v_{i}^{\prime })\;mod\;1,$$

(12) $$y_{i}=\{\begin{array}{c} 1,\;\;\;0\leq s_{i}<0.5\\ 0,\;\;\;0.5\leq s_{i}<1 \end{array}.$$

The NIST test standard includes 15 subsets. In the experiment, all subsets were considered, and each subset can output a *p*-value. If the *p*-value is greater than 0.01, the sequence is thought to pass a subset. The length of each binary sequence is 1,000,000 bits, and we test 100 binary sequences for each subset. During the process, the initial values of parameters for ESNDS are set as follows: γ=250, k=0.6, z=−0.1, h=0.01, and $u_{0}=0.1$. The result is shown in Table 2, and p−value of the last round is put into the table. According to [59], the minimum pass rate of each subset is 96 percent. Therefore, a dynamical system is chaotic enough if the minimum pass rate is achieved in all subsets.

#### 4.2.2. TestU01

To further investigate the pseudo-random sequence generated by ESNDS, two binary sequences are used in TestU01. As an empirical statistical test suite, TestU01 can evaluate the randomness of sequences through a collection of utilities [61]. The length of two binary sequences is 30,000,000 bits and 1,000,000,000 bits, respectively. In standard tests suits, the sequence size of nearly 30,000,000 is commonly used [62,63]. In the experiment, three predefined batteries, Rabbit, Alphabit, and Block Alphabit, are used to evaluate the randomness of bits generated by ESNDS. The initial values of parameters for ESNDS are set as follows: γ=250, k=0.6, z=−0.1, h=0.01, and $u_{0}=0.1$. The result is shown in Table 3. It can be seen that the sequences have strong randomness and ESNDS is effective.

#### 4.2.3. The Sensitivity to Initial Condition

**Figure 13:**
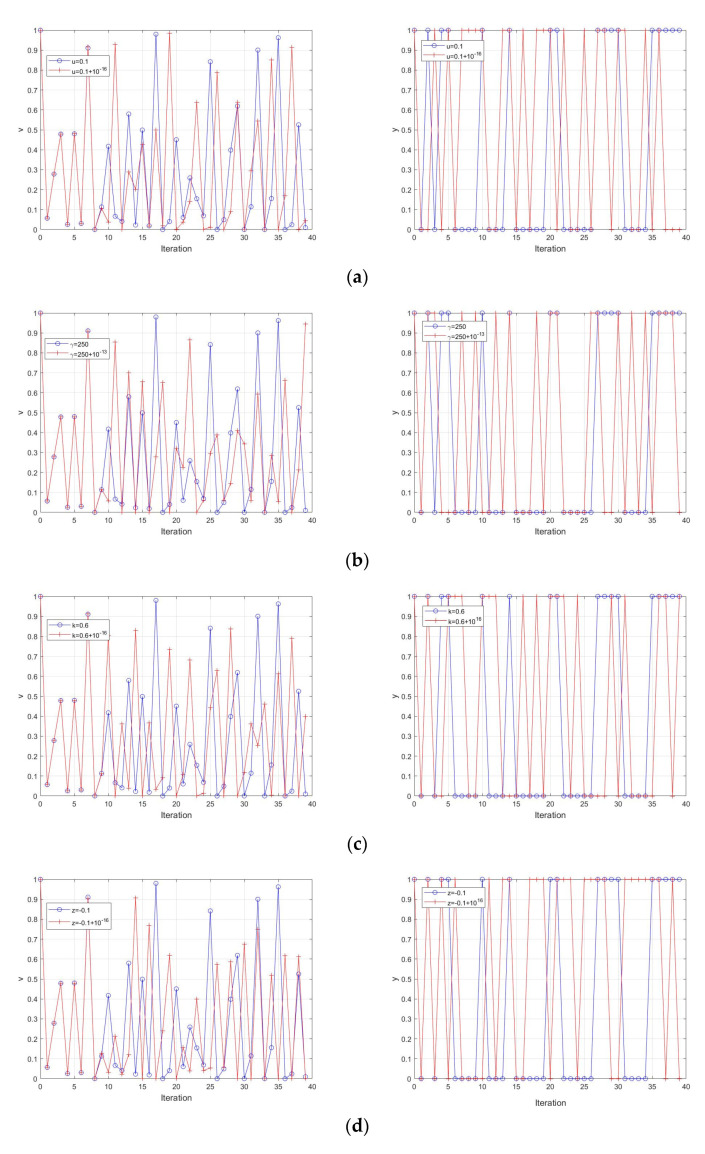

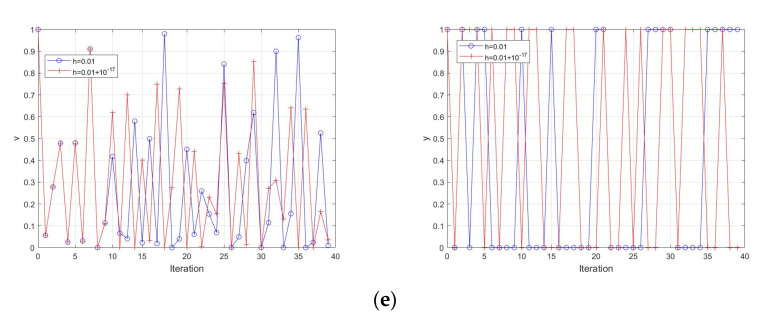
Sensitivity to initial condition of (**a**) varied initial value, for γ=250, k=0.6, z=−0.1, h=0.01; (**b**) varied γ, for $u_{0}=0.1$, k=0.6, z=−0.1, h=0.01; (**c**) varied k, for γ=0.6, $u_{0}=0.1$, z=−0.1, h=0.01; (**d**) varied z, for γ=0.6, $u_{0}=0.1$, k=0.6, h=0.01; (**e**) varied h, for γ=0.6, $u_{0}=0.1$, k=0.6, z=−0.1.

The sensitivity to initial condition is how slightly a parameter or initial value change will generate different sequence. In this section, the four parameters and initial value of ESNDS are studied. The result is shown in Figure 13. It is seen that the sequences vary at about ten iterations of all parameters and initial value. Therefore, ESNDS is sufficiently sensitive to initial condition and can fully ensure encryption security.

### 4.3. Performance Analysis

#### 4.3.1. Sample Entropy

Sample Entropy (SE) is used to describe the complexity of a time series quantitatively [64]. The computing method of SE is defined in [65]. The time series with a lower degree of regularity always have a larger SE. Therefore, a lager SE indicates that the time series is higher complexity. In order to reflect the complexity of the sequences generated by ESNDS clearly, we introduced two simple chaotic maps (i.e., Sine map, Logistic map) and Two coupled chaotic maps which are proposed in [48,55]. The coupled chaotic map in [48] is defined as Equation (13), and that in [55] is defined as Equations (14) and (15).

(13) $$\{\begin{array}{c} x_{i+1}=\sin\left(\pi r\left(y_{i}+3\right)x_{i}\left(1-x_{i}\right)\right)\\ y_{i+1}=\sin\left(\pi r\left(x_{i+1}+3\right)y_{i}\left(1-y_{i}\right)\right) \end{array},$$

(14) $$f\left(x\right)\{\begin{array}{c} \frac{x}{\alpha }\;\;\;\;0\leq x\leq \alpha \\ \frac{1-x}{1-\alpha }\;\;\alpha \leq x\leq 1 \end{array},$$

(15) $$x_{i+1}=f\left(4rx_{i}\left(1-x_{i}\right)\right).$$

**Figure 14:**
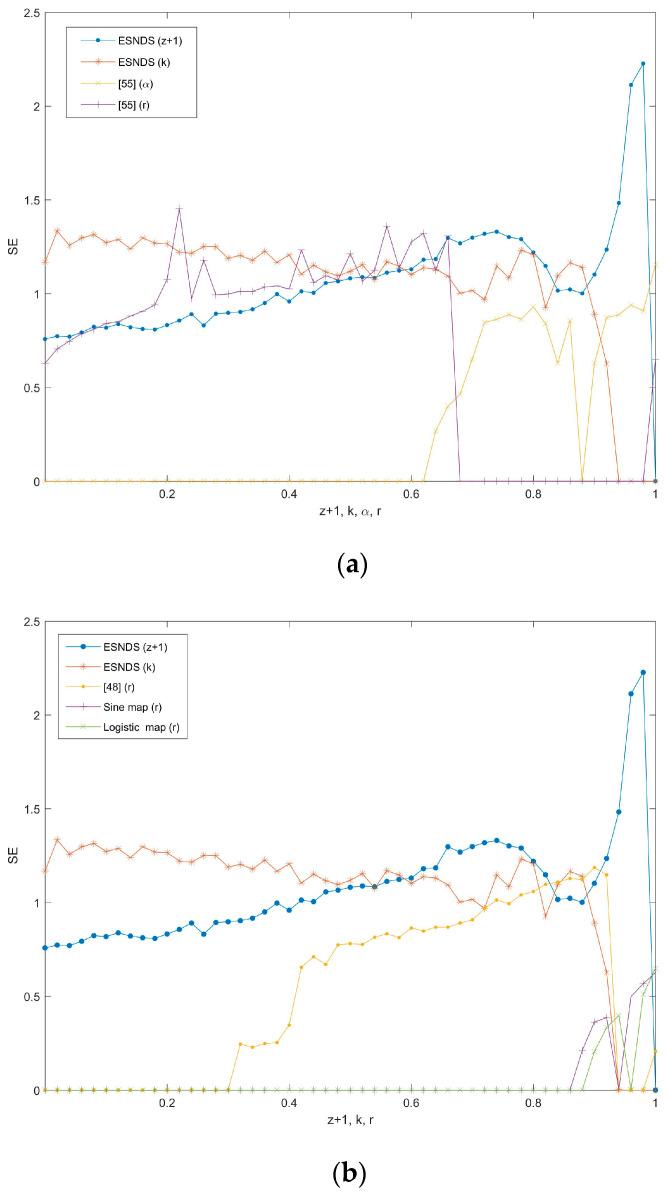
Comparison of SE between different maps: (**a**) ESNDS (z + 1) with γ=250, k=0.6, h=0.01, ESNDS (k) with γ=250, z=−0.1, h=0.01, [55] (α ) with r=4, and [55] (r ) with α=0.7; (**b**) ESNDS (z+1) with γ=250, k=0.6, h=0.01, ESNDS (k) with γ=250, z=−0.1, h=0.01, [48] (r), Sine map (r), and Logistic map (r).

For intuitive comparison, the parameters k∈[0,1] and z∈[−1,0] are selected to depict the SE of ESNDS, as shown in Figure 14. Furthermore, Figure 14a includes the SE of coupled chaotic map in [55] along parameters α and r, and Figure 14b includes the SE of coupled chaotic map in [48] and simple chaotic maps along parameter r. It can be seen that ESNDS have relatively wider chaotic range and larger SE than the simple chaotic maps and the coupled chaotic maps. This indicates that ESNDS can generate sequences with more complex properties. It is of significance for chaotic maps applied in data security.

#### 4.3.2. Efficiency Analysis

In considering the complexity of sequences generated by chaotic maps, the high efficiency of chaotic maps is also necessary. The implementation cost of ESNDS is calculated in different length of sequence, and it is also compared with coupled chaotic maps proposed in [48,55], as shown in Table 4. In the experiment, each sequence is generated 100 times, and the average running time is taken as the result. It can be seen that implementation cost of ESNDS is in the middle of the three chaotic maps. Therefore, ESNDS is suitable for data security.

## 5. Application to Image Encryption

### 5.1. Encryption Process

Step 1: The original grayscale image is read as a M×N matrix X for further processing. In addition, each element in the matrix is an integer from 0 to 255.

Step 2: The chaotic sequence is obtained from the ESNDS for encryption. $u_{0}$, γ, k, z and h are initial values of ESNDS, so they are used as the security keys. Iterate the ESNDS $\left(M\times N+M+N+U_{0}\right)$ times, and discard the former $U_{0}$ elements. Therefore, a new sequence with (M×N+M+N) is obtained.

Step 3: Take the former M elements as sequence a, the next N elements as sequence b, and the rest elements as sequence L. The following modifications were made to sequence a and b, as Equation (16):

(16) $$\{\begin{array}{c} a^{\prime }=floor\left(a\times M\right)+1\\ b^{\prime }=floor\left(b\times N\right)+1 \end{array}.$$

**Figure 15:**
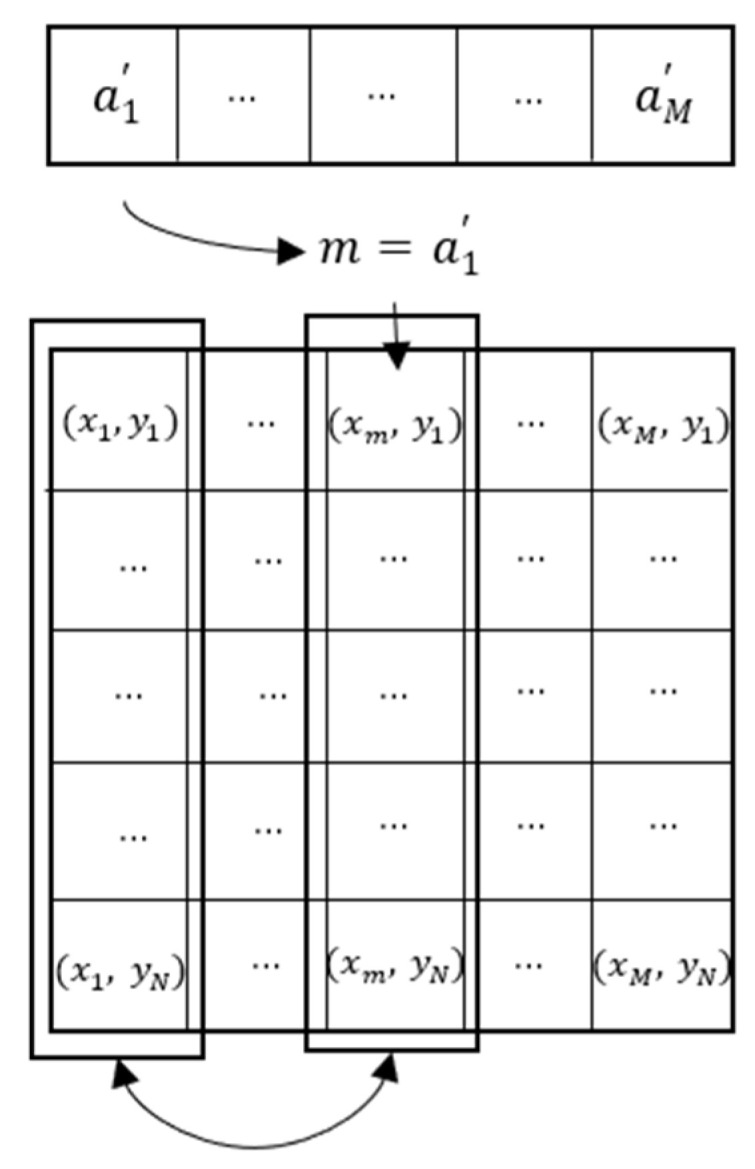
Matrix permutating process of column.

Step 4: Obtain the column permutation matrix. The process is shown in Figure 15.

**Figure 16:**
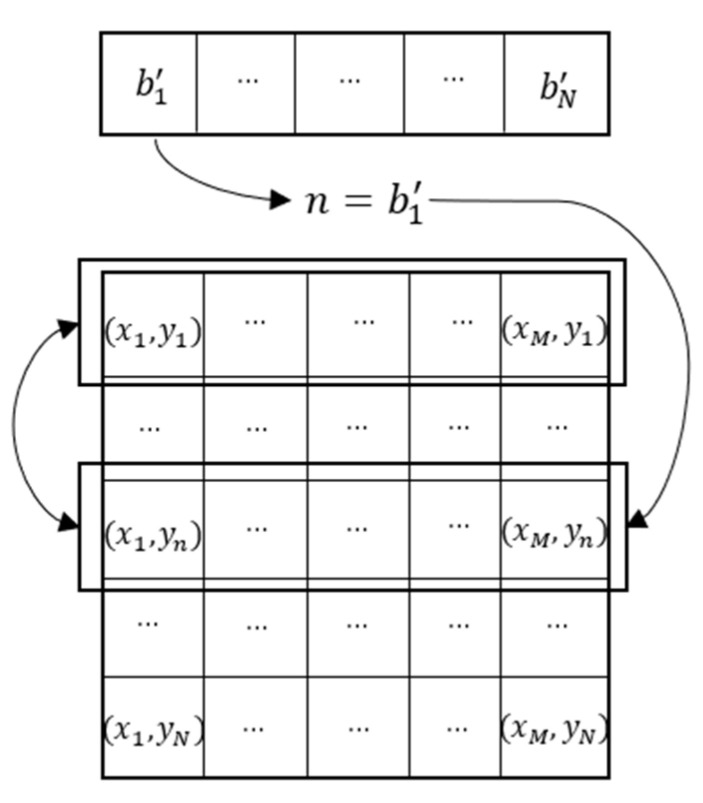
Matrix permutating process of row.

Step 5: Obtain the row permutation matrix. The process is shown in Figure 16.

**Figure 17:**
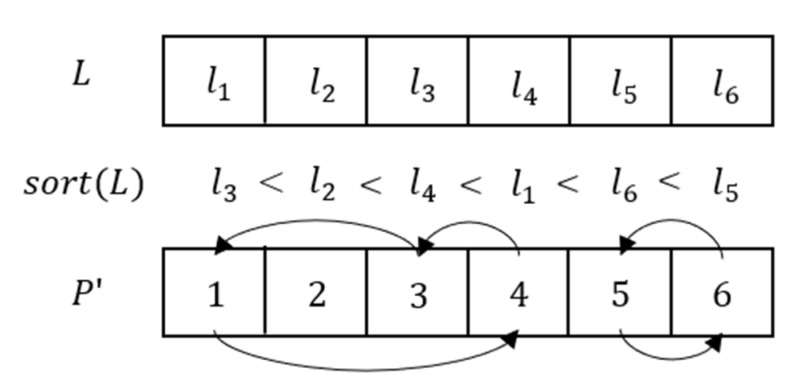
Permutating process of matrix P.

Step 6: The permutated matrix is converted into the 1D matrix $P=\left\{p_{1},p_{2},\ldots ,p_{M\times N}\right\}$, and sort the sequence L in ascending order. According to the sorting result, matrix $P^{\prime }$ is obtained. The process is shown in Figure 17.

Step 7: Obtain the diffused matrix H from the sequence L and the matrix $P^{\prime }$ by Equations (17) and (18):

(17) $$L^{\prime }=\left(floor\left(L\right)\times 10^{8}\right)\;mod\;256,$$

(18) $$H=P^{\prime }⨁L^{\prime }.$$

Step8: Convert H into the encrypted image with the size of M×N.

The decryption is the inverse process of encryption.

**Figure 18:**
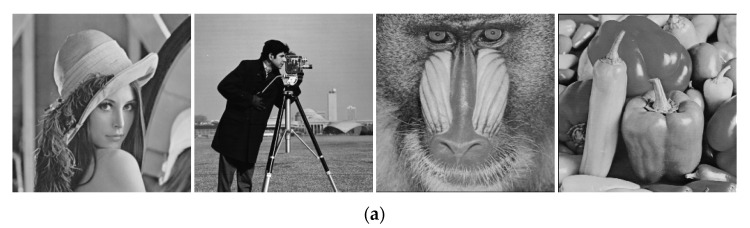

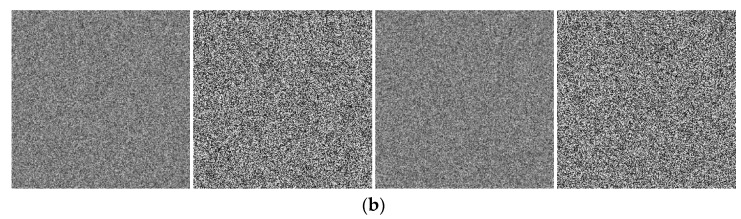
Encryption result of some images. (**a**) the original images; (**b**) the encrypted images.

In the experiment, the initial value of ESNDS $u_{0}=0.1$, the parameters γ=250, k=0.6, z=−0.1, h=0.01, and four images are used to verify encryption effect of the encryption method. The original images and results of encryption are shown in Figure 18.

### 5.2. Security Analysis

#### 5.2.1. Security Key Space

**Figure 19:**
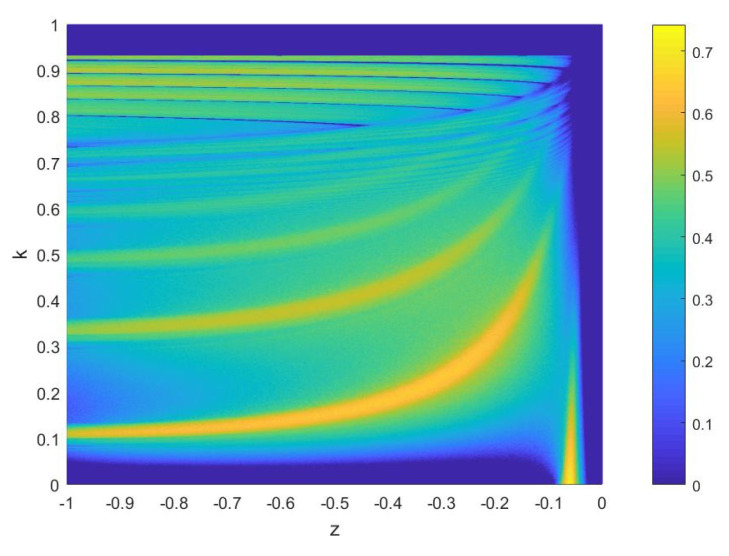
Two-dimensional evolution diagram of Lyapunov exponent (set Lyapunov exponent<0 to 0 ) of k−z for γ=250, h=0.01.

Key space refers to the summation of the different keys that can be used for encryption. Due to multiple parameters of ESNDS, it is very complicated to determine the range of all the keys that can generate chaotic sequences simultaneously. Therefore, we confirm the range of some parameters by the two-dimensional diagram of Lyapunov exponent to determine the minimum key space. The two-dimensional evolution diagram of Lyapunov exponent of k−z is shown as Figure 19. Figure 19 and Section 4.2.2 show that the both space of k and z is about $0.9\times 10^{16}$, and the space of $u_{0}$ is $1\times 10^{16}$. We can get the minimum key space is $0.9\times 10^{16}\times 0.9\times 10^{16}\times 10^{16}\approx 2^{162}$. The minimum key space is larger than $2^{128}$ which enough to resist brute force attacks [66,67].

#### 5.2.2. Information Entropy

The information entropy is a measurement standard of the degree of information ordering in digital images [68]. It is defined as Equation (19):

(19) $$H\left(x\right)=-\overset{n}{\underset{i=0}{\sum }}p\left(X_{i}\right)\log_{2}p\left(X_{i}\right)\;,$$

where n represents the grayscale level of an image, and $p\left(X_{i}\right)$ represents the probability of the grayscale value $X_{i}$. For a completely random image, the theoretical value of information entropy is 8 [69]. As shown in Table 5, the information entropy of encrypted images is close to the theoretical value. It shows the degree of information ordering tends to disorder after the encryption scheme.

#### 5.2.3. Correlation Analysis

**Figure 20:**
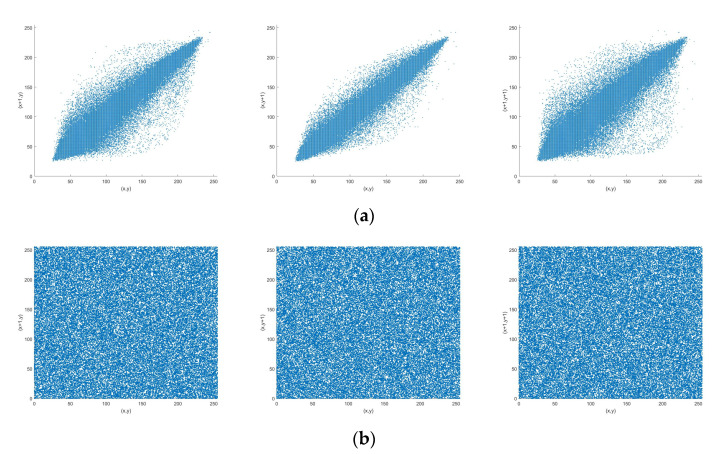
Correlation analysis of image Lena. (**a**) horizontal, vertical and diagonal correlation of original image; (**b**) horizontal, vertical and diagonal correlation of encrypted image.

In plaintext images, adjacent pixels tend to have high correlations. This is related to the discernibility of the information in the images. Therefore, it is necessary to reduce the correlation between adjacent pixels in the encrypted images [70]. The equation is shown as Equation (20):

(20) $$\{\begin{array}{c} \bar{x}=\frac{1}{N}\overset{N}{\underset{i=1}{\sum }}x_{i}\\ D\left(x\right)=\frac{1}{N}\overset{N}{\underset{i=1}{\sum }}x_{i}-\bar{x}\\ cov\left(x,y\right)=\frac{1}{N}\overset{N}{\underset{i=1}{\sum }}\left(x_{i}-\bar{x}\;\right)\left(y_{i}-\bar{y}\;\right)\\ \rho_{xy}=\frac{cov\left(x,y\right)}{\sqrt{D\left(x\right)}\sqrt{D\left(y\right)}} \end{array},$$

where, x and y are the gray values of adjacent pixels, and $\rho_{xy}$ represents the correlation coefficient between adjacent pixels. The horizontal, vertical and diagonal correlation of original image Lena and encrypted image Lena is shown in Figure 20. As shown in Table 6, compared with original images, the correlation coefficient of encrypted images is greatly reduced. This means that the encrypted images effectively conceal the information of the original images. In addition, Table 7 demonstrates the correlation coefficient of encrypted Lena using various encryption schemes. It can be seen that our scheme achieves relatively favorable performance among these methods.

#### 5.2.4. Sensitivity Analysis

A good encryption scheme should be sensitive to tiny changes in key and plaintext image. To test the sensitivity of the proposed scheme, two $u_{0}$ with only $1\times 10^{16}$ differences are used to encrypt the original images, respectively. The difference between two encrypted images can be measured through the Number of Pixel Change Rate (NPCR) and the Unified Average Changing Intensity (UACI). The NPCR and UACI are calculated by Equations (21) and (22) [72]:

(21) $$PCR=\frac{1}{M\times N}\sum_{i=1}^{M}\sum_{j=1}^{N}B\left(i,j\right)\times 100\%,$$

(22) $$UACI=\frac{1}{M\times N}\sum_{i=1}^{M}\sum_{j=1}^{N}\frac{P_{1}\left(i,j\right)-P_{2}\left(i,j\right)}{255}\times 100\%,$$

where $P_{1}$ and $P_{2}$ are two images with the size of M×N. If $P_{1}\left(i,j\right)\neq P_{2}\left(i,j\right)$, then B(i,j)=1, otherwise, B(i,j)=0. According to [77], the expected value of NPCR and UACI are 99.6094% and 33.4635% for 8-bit grayscale images. Table 8 shows the value of NPCR and UACI of four images. It can be seen that the proposed encryption scheme is sensitive to tiny changes in key.

#### 5.2.5. Histogram Analysis

**Figure 21:**
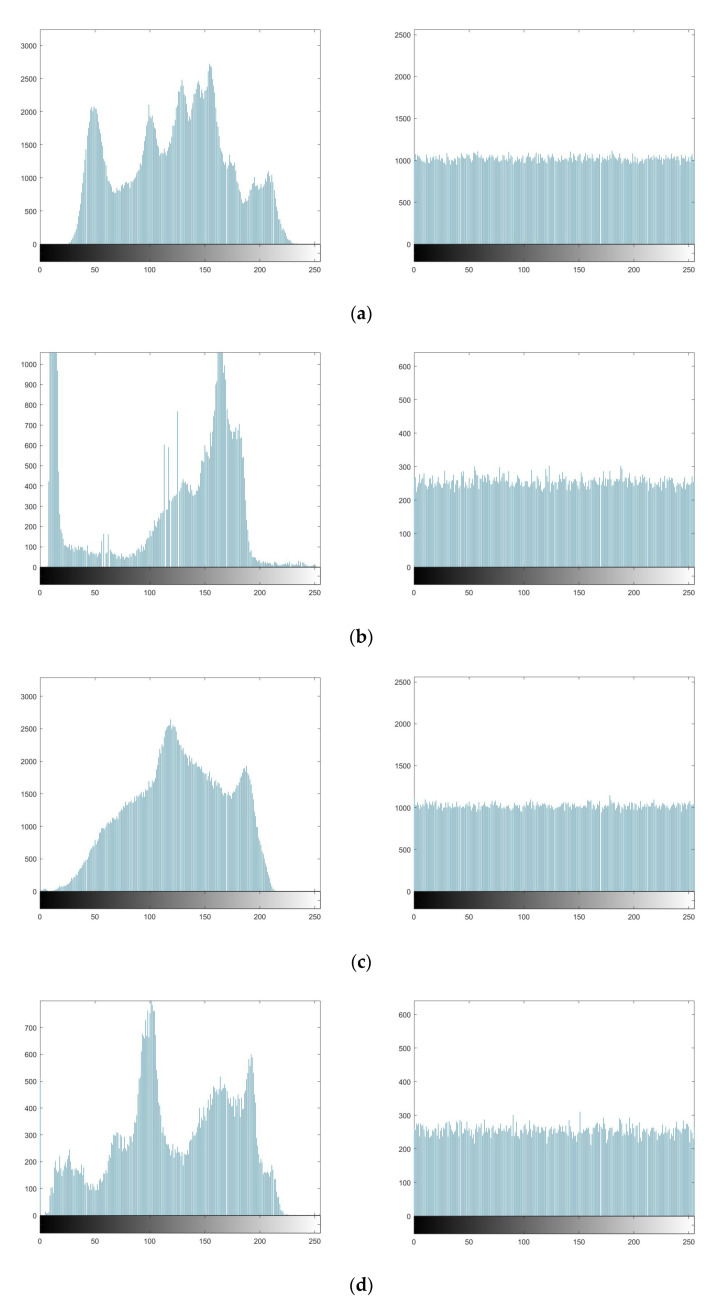
The histogram of original images and encrypted images versus (**a**) Lena; (**b**) Cameraman; (**c**) Mandrill; (**d**) Peppers.

The histogram analysis refers to the number of times each value appears, so as to reflect the distribution of pixel values of an image [21]. The ideal histogram should be flat and smooth to resist statistic attacks. The Figure 21 shows the histograms of four original images and the histograms of corresponding encrypted images. The pixel value distribution of the four encrypted images is uniform, so it can resist statistic attacks.

#### 5.2.6. Noise Robustness

Due to noise attack or noise jamming in the transmission channel, the pixel value modification of cipher images may appear [78,79]. The noise makes the information in cipher images difficult to recover. However, receivers would like to recover the original images as much as possible in the situation. Thus, an encryption scheme should have an ability of resisting noise.

**Figure 22:**
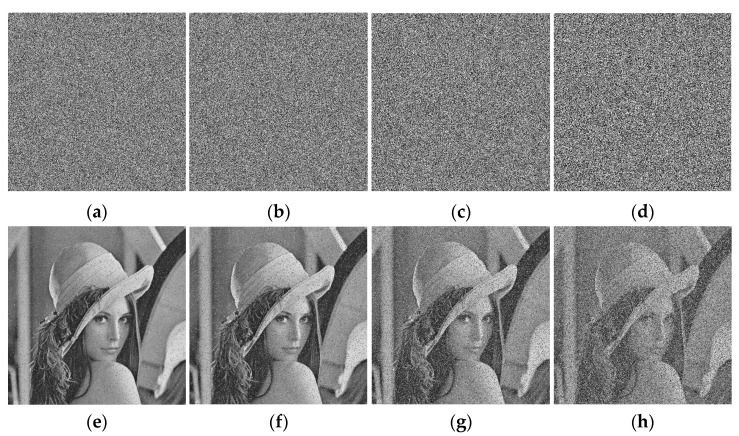
Noise analysis: (**a**) 5% ‘salt & pepper’ noise of encrypted Lena; (**b**) 10% ‘salt & pepper’ noise of encrypted Lena; (**c**) 25% ‘salt & pepper’ noise of encrypted Lena; (**d**) 50% ‘salt & pepper’ noise of encrypted Lena; (**e**) decrypted Lena from (**a**); (**f**) decrypted Lena from (**b**); (**g**) decrypted Lena from (**c**); (**h**) decrypted Lena from (**d**).

To test the ability of resisting noise, an experiment on noise attack is performed. Four different proportions of ‘salt & pepper’ noise are added to the encrypted Lena. The decryption process is then applied to the images with “salt & pepper” noise. The results are shown in Figure 22. It can be seen that the decrypted images recover most information of the original images.

#### 5.2.7. Robustness to Data Loss

In practical application, digital images are vulnerable to data loss in the process of communication for all kind of reason. This may be caused by the various interception, and some parts of digital images may be missing. In this case, the receiver can be easily failed to get the intact data. To cope with this, the encrypted images should have good anti-cutting performance.

**Figure 23:**
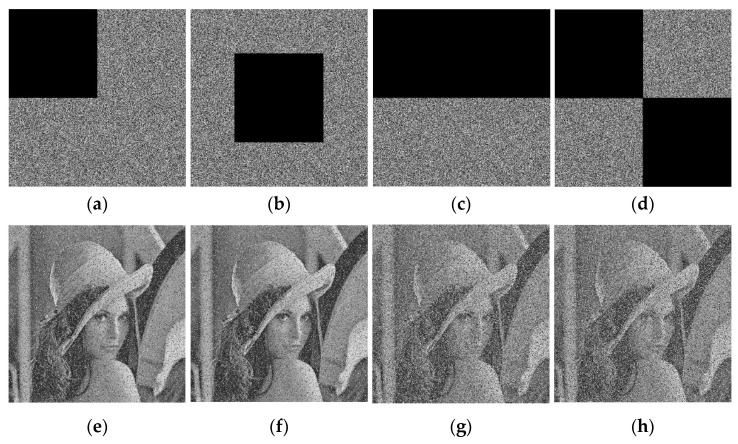
Data loss analysis: (**a**) 25% data loss of encrypted Lena; (**b**) 25% data loss of encrypted Lena; (**c**) 50% data loss of encrypted Lena; (**d**) 50% data loss of encrypted Lena; (**e**) decrypted Lena from (**a**); (**f**) decrypted Lena from (**b**); (**g**) decrypted Lena from (**c**); (**h**) decrypted Lena from (**d**).

Our proposed encryption scheme has enough robustness to data loss. The data loss is performed at the rate of 25% and 50% in different positions, and the processed images are used for decryption. The results are shown in Figure 23. It can be seen that the decrypted images restore most of the original details visually. This shows the encryption scheme has enough robustness to data loss.

### 5.3. Speed Analysis

Since the proposed encryption scheme is a kind of symmetric encryption scheme, the decryption is the inverse process of encryption. We only analyze the encryption speed in this section.

For the time complexity analysis of the scheme, the time-consuming part includes floating-point operations for the construction of chaotic sequences in ESNDS and permutation-diffusion process. Table 9 lists the computational complexity of the proposed encryption scheme as well as some other chaos-based image encryption schemes. The efficiency of the proposed scheme is comparable with existing chaos-based ciphers.

Furthermore, the speed of the encryption scheme is tested. The experimental environment is same as that in Section 3.2. The images with different size are encrypted, and the running time is shown in Table 10. In the experiment, each encryption is repeated 100 times, and the average running time is taken as the result. It can be seen that the average encryption/decryption speed of proposed scheme is enough for image encryption applications.

## 6. Conclusions

In this paper, the single neuronal dynamical system in self-feedbacked Hopfield network is proposed, and its derivation process of the discrete form is given. The chaotic dynamic behavior of the system is described from single-parameter and double-parameter perspectives. The implementation cost of the system is also lower than self-feedbacked Hopfield networks. Furthermore, we apply a framework for improving chaotic properties of the simple chaotic system to our system and achieve good performance. It is important to note that this applicability can make for the system being considered in more fields. In addition, an image encryption scheme based on the enhanced system is herein designed. The simulation results and security analysis prove that the scheme has an excellent performance.

The single neuronal dynamical system in self-feedbacked Hopfield Networks still has a large scope for exploration. In future work, we will continue improving the system, such as changing the activation function. 

## Figures and Tables

**Table 1:** Implementation cost (second) of SNDS and self-feedbacked Hopfield networks.

Length of Sequence	$1\times 10^{3}$	$1\times 10^{4}$	$1\times 10^{5}$	$1\times 10^{6}$	$1\times 10^{7}$
[21]	0.006050	0.013739	0.079042	0.635034	6.286282
[16]	0.005240	0.012485	0.071335	0.581641	5.802823
[19]	0.008347	0.020941	0.120890	0.971717	9.612208
SNDS	0.001287	0.003032	0.017690	0.163250	1.723895

**Table 2:** NIST SP800-22 test results of ESNDS.

Test Number	Subset	*p*-Value	Proportion	Test Result
1	Frequency	0.675947	100/100	Random
2	Block Frequency	0.124338	100/100	Random
3	Cumulative Sums	0.771002	100/100	Random
4	Runs	0.965044	99/100	Random
5	LongestRun	0.734606	98/100	Random
6	Rank	0.609329	100/100	Random
7	FFT	0.229310	99/100	Random
8	Non Over. Temp.	0.328353	100/100	Random
9	Over. Temp.	0.617757	100/100	Random
10	Universal	0.384464	98/100	Random
11	Appr. Entropy	0.663306	99/100	Random
12	Ran. Exc.	0.130397	99/100	Random
13	Ran. Exc. Var	0.341983	100/100	Random
14	Serial	0.320912	98/100	Random
15	Linear Complexity	0.340430	100/100	Random

**Table 3:** TestU01 test results of ESNDS.

Battery	Length of Sequences	Test Result
Rabbit	$3\times 10^{7}$	Pass
	$10^{9}$	Pass
Alphabit	$3\times 10^{7}$	Pass
	$10^{9}$	Pass
BlockAlphabit	$3\times 10^{7}$	Pass
	$10^{9}$	Pass

**Table 4:** Implementation cost (second) of ESNDS and different coupled chaotic maps.

Length of Sequence	$1\times 10^{3}$	$1\times 10^{4}$	$1\times 10^{5}$	$1\times 10^{6}$	$1\times 10^{7}$
[48]	0.001985	0.03982	0.023012	0.216749	2.118830
[55]	0.001984	0.003283	0.011455	0.083962	0.875654
ESNDS	0.001650	0.003364	0.019894	0.167419	1.730450

**Table 5:** Information entropy of different images.

Image	Lena	Cameraman	Mandrill	Peppers
Original image	7.4455	6.9719	7.3899	7.5327
Encrypted image	7.9993	7.9974	7.9993	7.9972

**Table 6:** Correlation coefficient of various images.

	Original Image			Encrypted Image		
Image	Horizontal	Vertical	Diagonal	Horizontal	Vertical	Diagonal
Lena	0.9850	0.9719	0.9593	0.0043	0.0018	0.0003
Cameraman	0.9592	0.9340	0.9089	0.0002	0.0067	0.0012
Mandrill	0.8003	0.8763	0.7627	0.0003	0.0014	0.0013
Peppers	0.9651	0.9759	0.9457	0.0019	0.0008	0.0069

**Table 7:** Correlation coefficient of various schemes.

	Encrypted Lena		
Scheme	Horizontal	Vertical	Diagonal
[46]	0.0024	−0.0086	0.0402
[49]	0.0021	0.0051	0.0040
[55]	0.0046	0.0063	0.0023
[56]	0.0013	0.0018	0.0032
[71]	−0.0084	−0.0017	−0.0019
[72]	0.0019	0.0038	−0.0019
[73]	0.0030	−0.0024	−0.0034
[74]	0.0013	−0.0141	−0.0054
[75]	0.0035	0.0065	0.0036
[76]	−0.0230	0.0019	−0.0034
Proposed	0.0043	0.0018	0.0003

**Table 8:** NPCR and UACI test result of different images ($u_{0}=0.1$ and $u_{0}^{\prime }=0.1+10^{-16}$ ).

Image	NPCR (%)	UACI (%)
Lena	99.6037	33.5093
Cameraman	99.6201	33.4603
Mandrill	99.6029	33.4717
Peppers	99.6201	33.4738

**Table 9:** Time complexity of different schemes.

Scheme	Time Complexity
Proposed	O(3MN+2M+2N)
[51]	O(8MN)
[71]	$O\left(18\mathrm{MN}+2\mathrm{MNlog}\frac{MN}{2}\right)$
[75]	O(9MN)
[76]	O(Mlog(8N)+8NlogM+M+8N)

**Table 10:** Encryption time of proposed scheme.

	Encryption Time (s)			
Image Size	128×128	256×256	512×512	1024×1024
Proposed scheme	0.021936	0.086149	0.355649	1.406963

## Data Availability

Not applicable.
